# Mediating Mechanisms in Cognitive Behavioral Therapy for Childhood OCD: The Role of Dysfunctional Beliefs

**DOI:** 10.1007/s10578-018-0830-8

**Published:** 2018-07-21

**Authors:** L. H. Wolters, P. J. M. Prins, G. J. A. Garst, S. M. Hogendoorn, F. Boer, L. Vervoort, E. de Haan

**Affiliations:** 1 Department of Child and Adolescent Psychiatry, Academic Medical Center, Meibergdreef 5, 1105 AZ Amsterdam, The Netherlands; 2 Academic Center for Child and Adolescent Psychiatry, De Bascule, Meibergdreef 5, 1105 AZ Amsterdam, The Netherlands; 3 Faculty of Social and Behavioural Sciences, University of Amsterdam, Nieuwe Achtergracht 127, 1018 WS Amsterdam, The Netherlands; 4 Department of developmental, personality and social psychology, Ghent University, Henri Dunantlaan 2, 9000 Ghent, Belgium

**Keywords:** Pediatric obsessive compulsive disorder, Cognitive behavioral therapy, Mediator of treatment, Obsessive beliefs, Cognitive theory

## Abstract

Reframing cognitions is assumed to play an important role in treatment for obsessive–compulsive disorder (OCD). However, there hardly is any empirical support for this assumption, especially for children. The aim of this study was to examine if changing dysfunctional beliefs is a mediating mechanism of cognitive behavioral therapy (CBT) for childhood OCD. Fifty-eight children (8–18 years) with OCD received CBT. Dysfunctional beliefs (OBQ-CV) and OCD severity (CY-BOCS) were measured pre-treatment, mid-treatment, post-treatment, and at 16-week follow-up. Results showed that OCD severity and dysfunctional beliefs decreased during CBT. Changes in severity predicted changes in beliefs within the same time interval. Our results did not support the hypothesis that changing dysfunctional beliefs mediates treatment effect. Future studies are needed to replicate these findings and shed more light on the role of explicit and implicit cognitions in treatment for childhood OCD.

## Introduction

The effectiveness of cognitive behavioral therapy (CBT) for childhood obsessive compulsive disorder (OCD) has been well established [1–5]. However, average symptom reduction is limited and almost half of the patients still have considerable complaints after standard treatment [6–8]. The way CBT is applied may not always be optimally effective [9–11].

CBT for OCD intends to change behavior (compulsions) and cognitions (obsessions). This is mostly done by a combination of exposure plus response prevention (ERP), and cognitive therapy (CT) [1]. Despite their common basis in the learning theory, distinct mechanisms leading to symptom reduction are assumed in ERP and in CT. From a behavioral perspective it is assumed that a behavioral change through exposure to feared situations is the primary process leading to essential corrective learning experiences. For this reason, behaviorists advocate ERP as the core of treatment [11–13]. In the cognitive model, it is assumed that the optimal way to corrective learning is through explicitly reframing dysfunctional beliefs. Therefore, in CT cognitive restructuring is advocated as the core of treatment [14, 15].

In line with the cognitive model, a relation between dysfunctional beliefs and obsessive–compulsive (OC) symptoms has been found in several child studies [16–25]. However, other studies yielded equivocal evidence for the cognitive model in childhood OCD [26–29]. In two studies it has been tested if inflated responsibility beliefs affected OC symptoms in children based on an experimental design. These studies yielded mixed results [24, 26]. Taken together, despite a well-developed theoretical base for a key role of dysfunctional beliefs in (childhood) OCD, the evidence is equivocal.

Nevertheless, cognitive models have strongly influenced treatment for OCD. Almost all treatment packages for childhood OCD contain some type of cognitive interventions [1, 2, 30, 31]. Full cognitive treatment protocols are developed even for children [32], and in combined treatment packages it is not unusual to start with a focus on cognitive interventions, followed by ERP. Efficacy of behavioral as well as cognitive oriented treatment protocols have been demonstrated [1, 33]. However, a favorable effect of ERP over CT has been reported [34], and the addition of cognitive interventions to ERP did not always result in more effective treatment packages [9, 35]. Overall, critiques on cognitive models are arising [35].

At present, the core of CBT is still unknown. Is explicitly changing cognitions the main mechanism through which symptom reduction is achieved, or would it be more effective to primarily focus on ERP?

A better understanding of the mechanisms of change in CBT may contribute to better treatment [1, 33]. Identification of these mechanisms can help to improve treatment because effective treatment components can be added or strengthened, and ineffective components can be removed [36]. However, mechanisms of change are rarely studied in treatments for childhood OCD.

Studying change mechanisms in treatment requires a formal test of statistical mediation. Mediation refers to the process through which change occurs. A change in the proposed mediator variable should precede a change in outcome. Consequently, a repeated measurements design is needed to demonstrate mediation [37]. Unfortunately, most treatment studies traditionally rely on pre-post test designs. An exception is a small pilot study [38]. In this study it was examined if changes in dysfunctional cognitions were associated with treatment effect in CT for pediatric OCD. Six adolescents with OCD (12–17 years, *M* = 14.3) reported responsibility beliefs and OCD symptoms at every treatment session. It was found that decreases in responsibility beliefs were associated with decreases in OCD symptoms, but the direction of this relation remained unclear, leaving the question of mediation unanswered [38].

Repeated measurements designs have been used slightly more often in adult OCD studies, with equivocal results. Whereas the findings of some studies provided some support for the cognitive model [39, 40], findings of other studies did not [34, 41–44], or were inconclusive [45, 46]. Most studies are based on small samples, and differences across studies in design and statistical analyses make it hard to draw clear conclusions. A preliminary observation is that most studies, including those with the largest sample sizes and based on thorough statistical analyses [34, 43, 44], did not support the cognitive mediation hypothesis.

Given the mixed evidence with regard to the potential role of dysfunctional beliefs in the treatment of childhood OCD, the aim of the present study was to further examine if changing dysfunctional cognitions is a mediator of treatment outcome in CBT for children and adolescents with OCD. It was examined whether changes in dysfunctional beliefs preceded changes in OCD severity, whether they were a consequence of changes in OCD severity, or whether this relation was bidirectional. Based on cognitive models it was our hypothesis that cognitive changes precede changes in OCD severity.

## Methods

### Design and Procedure

The present study was part of a trial intended to study psychological and neurobiological processes, non-response, and mediators of treatment outcome in childhood OCD. The trial was approved by the Medical Ethics Committee of the Academic Medical Center (MEC 06/053).^1^ The design and procedure of the study has been previously described in detail [8]. Participants were children and adolescents (8–18 years) who were referred for treatment for OCD to one of our treatment centers in the Netherlands. Inclusion criteria were a primary diagnosis of OCD according to *The Diagnostic and Statistical Manual of Mental Disorders* (4th ed., text revision; DSM-IV-TR) criteria, OCD complaints for at least 6 months, and a minimum score of 16 on the Children’s Yale-Brown Obsessive Compulsive Scale (CY-BOCS). Exclusion criteria were medication for OCD (SSRI, tricylic antidepressants or antipsychotic medication), CBT for OCD during the past 6 months, IQ below 80, and psychosis. Informed consent was obtained from all individual participants included in the study. Next, participants were randomly assigned to CBT or waitlist followed by CBT. CBT did not differ across conditions.

Assessments were conducted pre-treatment (T1), mid-treatment (T2; session 8), post-treatment (T3; session 16) and at 16 weeks follow-up (T4). During the assessments, the Children’s Yale-Brown Obsessive Compulsive Scale (CY-BOCS; see below) and the Obsessive Belief Questionnaire—Child Version (OBQ-CV; see below) were administrated. Additional assessments and measures for the purpose of the full trial are not described here. Participants received a small compensation for their efforts after completing the assessments (i.e., a gift voucher with a value of 10 Euro for each assessment).

### Participants

**Fig. 1:**
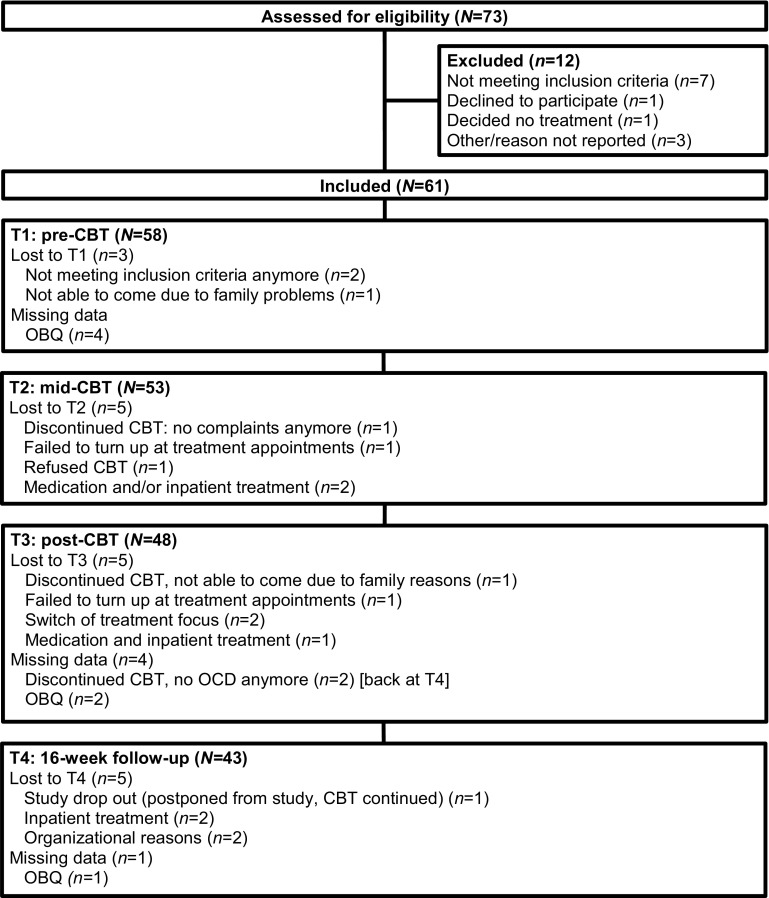
Flow chart

Between January 2007 and June 2010, 73 children were screened for eligibility in the study and 61 children (84%) were included. Three children dropped out before the pre-treatment assessment (T1): two did not meet the inclusion criterion anymore, and one child was not able to visit the clinic due to family circumstances. Finally, 58 children were included. Forty-six of the 58 children (79%) completed the CBT. Reasons for drop out were: early termination of treatment because complaints were in remission (*n* = 3), OCD symptoms were in remission before session 16 and treatment switched to other problems (*n* = 2), patients were unable or unwilling to visit the clinic (*n* = 4), referral to inpatient treatment for OCD and/or co-morbid problems (*n* = 2), and addition of medication (*n* = 1). Forty-eight children completed the post-CBT (T3) assessment, and 43 children completed the 16-week follow-up (T4) assessment (see Fig. 1 for the flow chart). The trial was ended because the intended number of participants was reached. No adverse events were reported.

Demographic and clinical characteristics of the sample have been previously described in more detail [8], and are summarized in Table 1.

**Table 1:** Baseline characteristics of participants (*N* = 58)

Age	*M* (SD)	12.8 (2.6)
Gender	Boys	24 (41.4%)
	Girls	34 (58.6%)
Cultural background	Dutch	46 (79.3%)
	Non-western	3 (5.2%)
	Other/combined	4 (6.9%)
	Missing	5 (8.6%)
CY-BOCS	*M* (SD)	24.8 (4.1)
	range	16–35
ADIS	Any co-morbidity	38 (65.5%)
	Anxiety disorder	31 (53.4%)
	Mood disorder	9 (15.5%)
	ADHD/ODD	9 (15.5%)

### Treatment

Treatment consisted of 16 weekly sessions of manualized individual CBT described in the Dutch treatment manual ‘Bedwing je dwang’ (‘Control your OCD’) [47]. The treatment involves psychoeducation, building a hierarchy of OC symptoms, exposure with response prevention (ERP), cognitive interventions (CI), and relapse prevention. ERP is introduced early in treatment (second session), followed by and combined with cognitive interventions (start at second or third session). ERP involved therapist-assisted practice during the sessions and (daily) exercises at home. Cognitive treatment contained a basic part (information about cognitions and their relation with feelings and behavior, and about the role of cognitions in OCD), and a range of additional interventions varying in complexity from simple (for example, replacing dysfunctional thoughts by thoughts that help to cope with anxiety or distress) to more sophisticated cognitive techniques (for example, probability estimates, and listing pros and cons) in order to enable therapists to tailor treatment to the individual. Guidelines for the selection of cognitive interventions, based on age, intellectual level, interest/motivation, and insight in their complaints, were provided in the manual. All participants received the basic part and one or more additional cognitive interventions during treatment. Treatment sessions lasted 45–60 min. Parents were involved in the therapy. CBT was delivered by master level clinicians certified as cognitive behavioral therapists and experienced in treating OCD in children. Therapists were trained in the protocol and attended group supervision every 2 weeks combined with optional individual supervision by the last author.

### Treatment Adherence

To allow for the assessment of treatment adherence, therapists completed a report of each treatment session. Twenty-five percent of the session reports of each participant were evaluated by two independent raters. Criteria for adequate treatment adherence were: psychoeducation and building a hierarchy of OC symptoms during the first session, ERP and/or CI and homework exercises during session 2–15, and relapse prevention in the last session. For 98.5% of the sessions criteria for adequate treatment adherence were met. Raters agreed for 99% of the session reports, Cohen’s kappa was 0.75.

### Measures

The *Anxiety Disorder Interview Schedule* for DSM-IV–Child and Parent Version (ADIS-C/P) [48, 49] is a semi-structured interview evaluating prevalence and severity of DSM-IV diagnoses of anxiety disorders, mood disorders, ADHD and disruptive disorders. The ADIS-C/P has demonstrated good to excellent test–retest and interrater reliability, and adequate concurrent validity [50, 51]. A clinician severity rating (CSR, ranging from 0 to 8) of at least four is indicative of a diagnosis. The combined CSR was used, which means that in cases in which the child and parent interviews yield the same diagnosis, the higher of the two severity ratings was assigned. Interviews were videotaped and 19 interviews (random selection) were independently rated by two raters to examine inter-rater reliability. Cohen’s kappa was 0.93.

The *Children’s Yale-Brown Obsessive Compulsive Scale* (CY-BOCS) [52] severity scale is a clinician-rated semi-structured interview evaluating the severity of OC symptoms, and consists of an obsession and a compulsion subscale. Each subscale contains 5 items assessing frequency/time, interference, distress, resistance, and control. Items are rated by the clinician on a 5-point scale from 0 to 4. The total score, the sum of both subscales, ranges from 0 to 40. A total score of 16 or more is considered as clinically significant [6]. The CY-BOCS demonstrated good reliability and adequate divergent and convergent validity [52]. Cronbach’s *α* in the present study ranged from 0.81 to 0.96. To examine inter-rater reliability, interviews were videotaped and 46 interviews (random selection) were independently rated by three raters (investigators and therapists). The intraclass correlation coefficient was 0.98.

The Dutch version of the *Obsessive Belief Questionnaire–Child Version* (OBQ-CV) [28] was used. The OBQ-CV is a self-report questionnaire about OCD-related dysfunctional beliefs, consisting of three subscales (44 items): Responsibility/Threat Estimation (RT), Perfectionism/Certainty (PC), and Importance/Control of Thoughts (ICT). Answers are scored on a five-point scale, ranging from 1 (never) to 5 (always), with higher scores indicating more obsessive beliefs. A study on psychometric properties of the Dutch version of the OBQ-CV in a community sample (*N* = 547; 8–18 years) and an OCD sample (*N* = 67; 8–18 years) yielded support for its reliability and validity. Cronbach’s alpha was 0.95, and retest reliability was adequate. Confirmatory factor analyses revealed best fit for a four-factor model representing Perfectionism/Certainty, Importance/Control of Thoughts, Responsibility, and Threat, and a higher-order factor [28]. Cronbach’s α (T1–T4) in the present study ranged from 0.96 to 0.98.

### Statistical Analyses

The intention-to-treat principle was used for the analyses, unless otherwise mentioned. For missing data at the level of missing items, missing values on the OBQ-CV were replaced by the individual mean of all valid items. In case of more than 5 missing items the OBQ-CV was considered as missing completely. Cases with missing measures or assessments were compared to complete cases on age, gender, pre-treatment OCD severity (CY-BOCS) and pre-treatment dysfunctional beliefs (OBQ). There were no significant differences between groups. Patterns of missing data were inspected using the Missing Value Analysis (MVA) output and Little’s Missing Completely At Random (MCAR) test in SPSS. Little’s MCAR test did not reach significance. We found no evidence that the missingness of data were related to any variable in this study. Missing assessments (OBQ-CV, CY-BOCS) were imputed using the expectation–maximization (EM) algorithm in LISREL version 8.8. Fifty-eight participants were included in the analyses.

#### Effect of Treatment

A prerequisite for a mediation analysis is an effective treatment. To test whether this condition was met, we performed a linear mixed model analysis in SPSS with time (T1–T4) as independent variable and OCD severity (CYBOCS) as dependent variable. This analysis was performed with both an unstructured covariance matrix and an autoregressive heterogeneous matrix. Fit of both models were compared using the − 2 log likelihood values. As results revealed no significant difference in fit between models, preference was given to the autoregressive heterogeneous matrix as this matrix provided a more parsimonious model.

#### Mediation Analysis

A series of latent different score (LDS) models was used to examine if changes in dysfunctional beliefs mediated treatment outcome [53]. Analyses were conducted with LISREL version 8.8. All models were estimated using maximum likelihood.

**Fig. 2:**
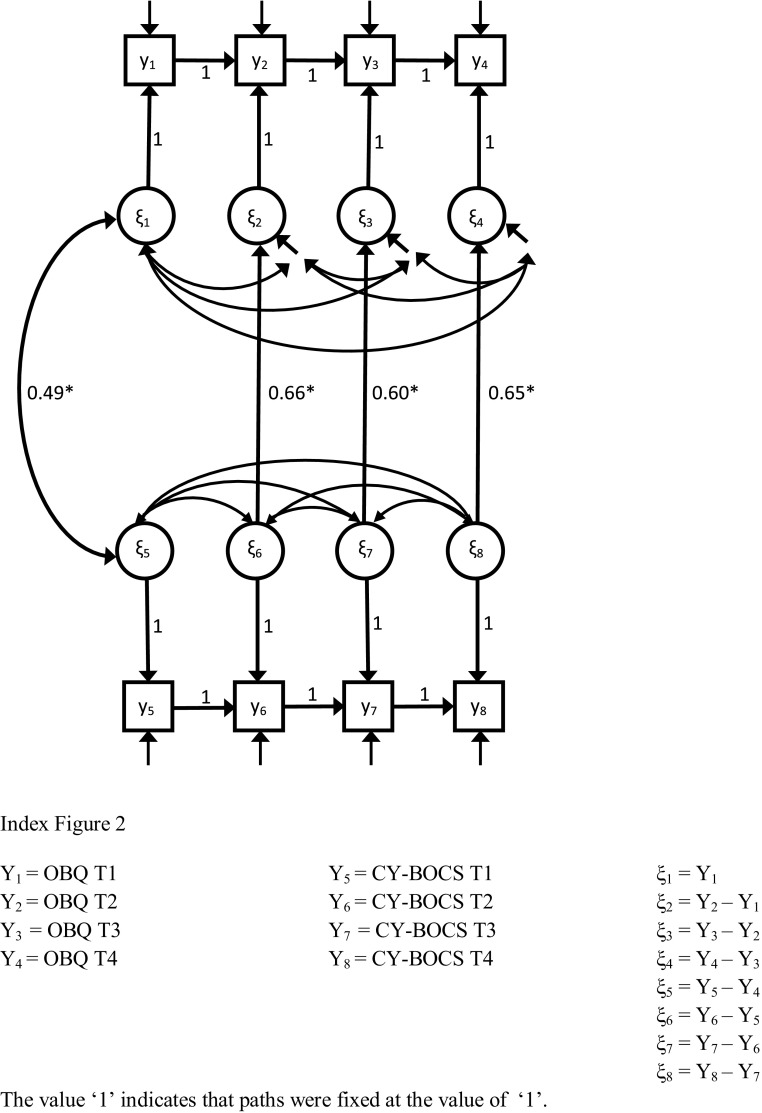
Schematic representation of the LDS model

LDS models are recommended for examining within-individual change over time in situations in which change may not be constant for each interval in the model [54]. The latent difference score (or ‘gain score’) variables in these models represent the difference between two successive latent true scores. Latent true score variables are composed of the preceding latent true score and an accumulation of latent changes over time (see Fig. 2). In addition, LDS models allow for examination of within-individual change as well as individual differences in the within-individual change [54, 55]. An advantage of LDS models relative to other models that could be used to examine change over time (e.g., latent growth curve (LGC) models) is that trajectories over time are free to vary, which means that there are no restrictions on the nature of change over time. In contrast to autoregressive (AR) models, changes are not restricted to residual changes. The dynamic latent difference score model [56] was also taken into consideration. However, in these models change scores are composed of a systematic trend-like component (as in LGC models), and a component explained by the previous assessments (as in AR models). Both components impose restrictions on change trajectories over time.

In conclusion, LDS models allow for testing dynamic relationships between dysfunctional beliefs and OCD severity without imposing a particular change mechanism on the data. As we had no specific hypothesis concerning the change trajectories over time of both variables, we preferred the LDS model over the Dynamic LDS Model to study the dynamic interplay between changes in dysfunctional beliefs and OCD severity.

To restrict the number of parameters to be estimated in the LDS models, we used the total scores of the OBQ-CV and the CY-BOCS as single indicators, instead of using subscale or item scores. We corrected for measurement error by fixing the error variances to values that were based on variances and the reliabilities of the scales.^2^ Reliability was based on Stratified Alpha, which is recommended for composite scales [58, 59]. Figure 2 shows a schematic picture of the LDS model.

**Fig. 3:**
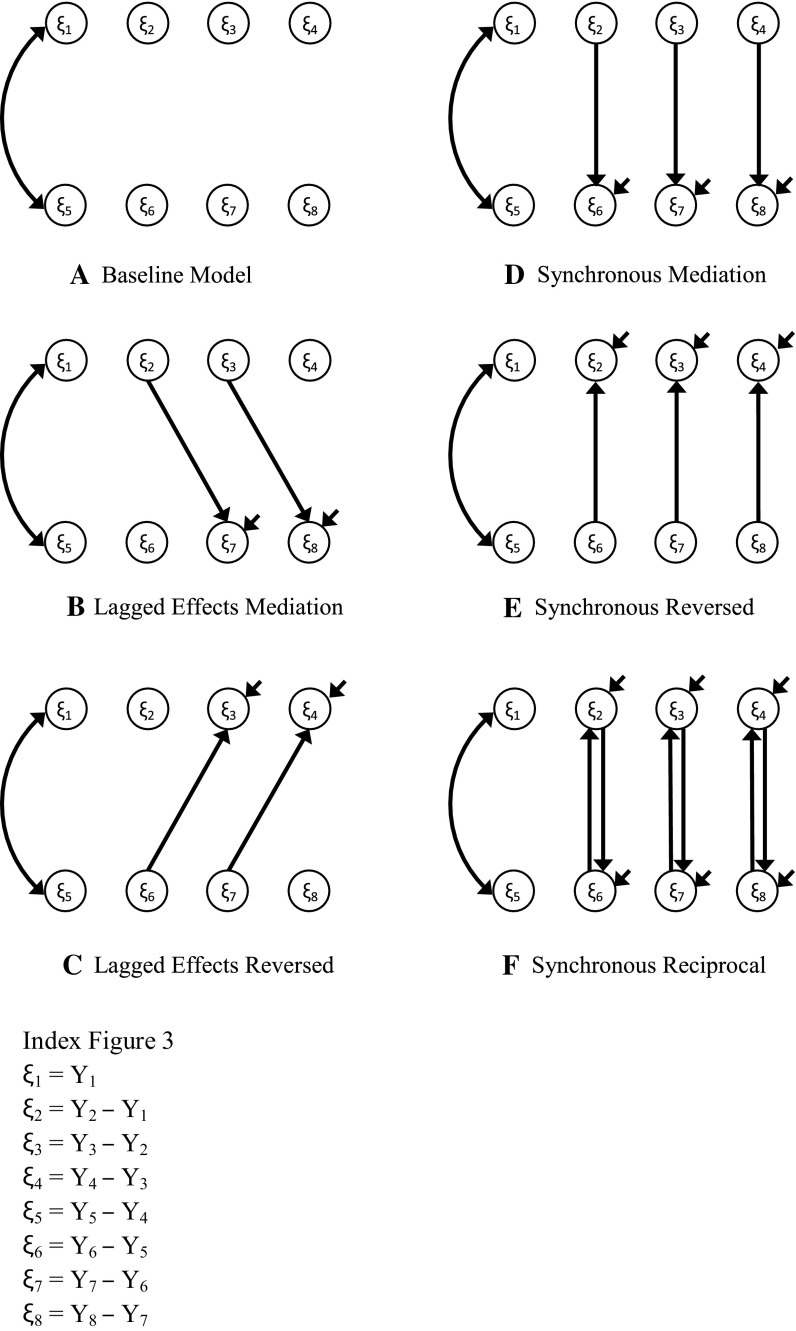
Schematic representation of LDS models

Several hypotheses were examined using multivariate LDS models. For both the OBQ-CV and the CY-BOCS four time points were included: pre-treatment (T1), mid-treatment (T2), post-treatment (T3), and follow-up (T4). We started with a Baseline Model in which latent changes between dysfunctional beliefs and OC symptoms were unrelated (see panel a in Fig. 3). When the Baseline Model is rejected, several hypotheses can be tested concerning the relations between changes in the both constructs. First, we tested whether changes in dysfunctional beliefs preceded changes in OCD severity (Lagged Effects Mediation Model). In this model, lagged effects between dysfunctional beliefs at time T on OCD severity at time T + 1 were specified (see panel b in Fig. 3). Second, we tested for the reversed effect: whether changes in dysfunctional beliefs at time T + 1 were the result of changes in OCD severity at time T (Lagged Effects Reversed Model; see panel c in Fig. 3). Third, because intervals between successive time points were relatively long (e.g., 8 weeks), we also tested a synchronous model. In this model, which is referred to as Synchronous Mediation Model, effects of changes in dysfunctional beliefs at time T on changes in OCD severity at time T were specified (see panel d in Fig. 3). This model implies that effects have occurred somewhere between time T and the previous assessment. The time interval between cause and effect is shorter than the interval between two successive measurement time-points [60]. Synchronous models provide weaker support for mediation than lagged models as the assumption of time precedence for cause–effect relations is not met. We also tested the reversed effect: whether changes in OCD severity predicted changes in beliefs within the same time interval (Synchronous Reversed Model; see panel e in Fig. 3). Finally, we tested whether there was a bi-directional relation between changes in beliefs and changes in OCD severity (Synchronous Reciprocal Model; see panel f in Fig. 3).

The following fit indices were selected to evaluate model fit: the minimum fit function Chi square statistic [61], the Akaike Information Criterion (AIC), the root mean square error of approximation (RMSEA), and the Comparative Fit Index (CFI). Low Chi square and AIC values, RMSEA values below 0.06, and CFI values above 95 are generally assumed to indicate good model fit [62, 63]. The Chi square difference test (the change of *χ*^2^ relative to the change in degrees of freedom) was used to test whether an alternative model leads to a significant improvement with regard to the original model [63].

#### Sample Size

There are no clear guidelines for the required sample size in structural equation modeling, as sample size requirements are affected by many factors, such as model complexity and estimation algorithm [63]. In the LDS model, most parameters are simple transformations of the covariances of the observed variables. The number of additional structural parameters that were estimated in the LDS model varied between 3 and 6. Given the low number of structural parameters, the present sample size (*N* = 58) is considered acceptable.

## Results

### Effect of Treatment

To test whether the prerequisite of an effective treatment was met, we first examined whether there was a decrease of OCD severity during (and following) treatment. Results of the linear mixed model analysis revealed a main effect of time (T1–T4) on CY-BOCS score, F(3, 45.88) = 78.72, p < 0.001. Table 2 shows the parameter estimates for each measurement compared to baseline (T1). CY-BOCS scores significantly decreased during CBT, there was a trend for a decrease until 16 weeks follow-up. These results suggest that the present CBT was effective.

**Table 2:** CY-BOCS: parameter estimates compared to baseline (T1)

	b	SE b	95% CI	Pairwise comparisons
T2 (8 sessions CBT)	− 6.26	0.84	− 7.94; − 4.56	T2 < T1 (p < 0.001)
T3 (16 sessions CBT)	− 12.92	1.18	− 15.30; − 10.54	T3 < T2 (p < 0.001)
T4 (16 weeks follow-up)	− 14.67	1.00	− 16.68; − 12.65	T4 < T3 (p = 0.10)

### Mediation Analysis

Table 3 shows the means, standard deviations, and correlations between the CY-BOCS and OBQ-CV scores for all assessments (missing values imputed).

**Table 3:** Means, standard deviations and correlations between CY-BOCS and OBQ-CV scores

	*M*	SD	Pearson correlation (*r*)							
			CB 1	CB 2	CB 3	CB 4	OBQ 1	OBQ 2	OBQ 3	OBQ 4
CB 1	24.71	5.04								
CB 2	18.43	5.93	0.41**							
CB 3	11.90	8.86	0.51**	0.76**						
CB 4	10.10	6.43	0.41**	0.68**	0.72**					
OBQ 1	107.76	29.85	0.44**	0.38**	.25^t^	0.30*				
OBQ 2	100.76	33.65	0.33*	0.52**	38**	0.37**	0.88**			
OBQ 3	91.90	34.46	0.45**	0.63**	0.62**	0.50**	0.75**	0.85**		
OBQ 4	93.40	30.99	0.35**	0.53**	0.41**	0.44**	0.79**	0.87**	0.89**	

CB = CY-BOCS, 1 = T1, 2 = T2, 3 = T3, 4 = T4

*Correlation is significant at the 0.05 level, **Correlation is significant at the 0.01 level

Figure 2 shows a simplified schematic version of each LDS model that was fitted to examine (temporal) relations between changes in dysfunctional beliefs (OBQ-CV) and OCD severity (CY-BOCS) during CBT and follow-up. Table 4 shows fit indices for all models.

**Table 4:** Fit indices

Model	χ^2^ (*p* value)	df	RMSEA	90% CI RMSEA	close fit RMSEA *p* value	AIC	CFI
(1) Baseline model	54.38 (< 0.001)	15	0.18	0.12–0.24	0.001	84.37	0.92
(2) Lagged effects mediation	52.81 (< 0.001)	13	0.20	0.14–0.27	< 0.001	88.75	0.92
Difference model 2 versus model 1	1.57 (0.46)	2					
(3) Lagged Effects Reversed	48.85 (< 0.001)	13	0.19	0.13–0.26	< 0.001	86.80	0.93
Difference model 3 versus model 1	5.53 (0.06)	2					
(4) Synchronous mediation	20.77 (0.05)	12	0.11	0.00–0.19	0.14	67.72	0.98
Difference model 4 versus model 1	33.61 (< 0.001)	3					
(5) Synchronous reversed	9.02 (0.70)	12	0.00	0.00–0.10	0.81	56.75	1.00
Difference model 5 versus model 1	45.35 (< 0.001)	3					
(6) Synchronous reciprocal	4.19 (0.90)	9	0.00	0.00–0.06	0.93	58.17	1.00
Difference model 6 versus model 5	4.83 (0.19)	3					

The Baseline Model (Model 1 in Table 4) in which changes in dysfunctional beliefs and changes in OCD severity were unrelated, did not fit the data as indicated by a high and significant Chi square and the high values for the AIC and RMSEA. Therefore, we tested a series of models that specified several possible relations between changes in beliefs and OCD severity. The Lagged Effects Mediation Model (Model 2) did not significantly improve model fit, as indicated by the non-significant Chi square difference test (Δχ^2^ = 1.57, df = 2, p = 0.46), and the high value of the RMSEA. The Lagged Effects Reversed Model (Model 3), testing the alternative hypothesis that a change in beliefs was the result of a change in OCD severity also showed inadequate fit indices. The inadequate fit of the lagged effects models could be explained by the relatively long time interval between assessments. Therefore, we estimated a series of synchronous models which allow shorter time intervals between cause and effect. The synchronous models (Models 4, 5) fitted the data significantly better than the Baseline Model (indicated by significant Chi square difference tests), and showed acceptable fit indices. Best model fit was found for the Synchronous Reversed Model (Model 5) which showed excellent fit values on all goodness of fit measures (low and non-significant Chi square value, RMSEA < 0.05, and CFI > 0.95), and the lowest value of the AIC. The Synchronous Reciprocal Model (Model 6) is nested in the Synchronous Reversed Model. Model 6 did not show significant improvement to Model 5 as indicated by the non-significant Chi square difference test, and three path coefficients were non-significant (all reflecting an effect of the OBQ on the CY-BOCS). Therefore, this model was rejected.

The best fitting model was the Synchronous Reversed Model. Results for this model showed significant, positive effects of the CY-BOCS on the OBQ at each assessment point. Parameter estimates were 0.18 (SD 0.04) for ∆CY-BOCS_T1−T2_ → ∆OBQ_T1−T2_; 0.20 (SD 0.04) for ∆CY-BOCS_T2−T3_ → ∆OBQ_T2−T3_; and 0.16 (SD 0.03) for ∆CY-BOCS_T3−T4_ → ∆OBQ_T3−T4_. Squared multiple correlations were 0.43; 0.36; and 0.42 respectively (see Fig. 2 for this model).

## Discussion

The aim of the present study was to examine if changing dysfunctional beliefs was a mediator of CBT for pediatric OCD. Fifty-eight children with OCD (8–18 years old) received sixteen weekly sessions of CBT consisting of ERP and CT. Dysfunctional beliefs and OCD severity were assessed pre-treatment, mid-treatment, post-treatment, and at 16-week follow-up. According to cognitive models, we expected cognitive changes to precede changes in OCD severity. Contrary to this hypothesis, results showed that changes in OCD severity statistically predicted changes in dysfunctional beliefs rather than the reverse. In other words, changes in severity explained changes in dysfunctional beliefs within time intervals. More specifically, changes in OCD severity explained 43% of the change in beliefs at mid-treatment, 36% between mid- and post-treatment, and 42% between post-treatment and follow-up. It is important to note that the present results do not allow for conclusions about causality, because we did not find a relation between cognitions and OCD severity over time (across assessment points). Therefore, we cannot determine whether a decrease in dysfunctional beliefs actually was an *effect* of a decrease in OCD severity. Nevertheless, the present findings cast doubt on the assumption of cognitive models suggesting that changing beliefs plays a key role in the treatment of OCD.

In line with the present results, several previous studies evaluating mediating mechanisms in psychological treatment for OCD were not supportive for cognitive models [34, 41–44]. However, this field of research is still in its infancy and is hampered by the limited amount of studies, the main focus on adult samples, and methodological limitations and differences across studies. There are some other issues that merit discussion.

Studying cognitive mediation is a challenging task. One of the biggest challenges is to measure actual thought processes. Cognitive processes may rely on conscious thoughts as well as on unconscious, automatic thoughts. Explicit measures, either standardized or idiographic, can only provide indications of beliefs that are accessible for personal introspection. In addition to explicit measures, implicit paradigms may be needed to shed light on the role of unconscious, automatic thoughts [64, 65]. Although still in its early stages, several cognitive bias paradigms have been developed for this purpose [66]. It would be interesting to use such paradigms in future studies. It could be for example, that contrary to the explicit approach in cognitive therapy, ERP addresses implicit cognitive processes. Implicit paradigms may be more suitable to detect these mechanisms.

Another challenge is that mediation requires a relation between the mediating variable and the outcome variable over time [37]. One could wonder if—even in case of established cognitive mediation—the mediating processes can be disentangled over time. One may assume that as soon as thought processes change, for example the patient does not overestimate the importance of an intrusion anymore, no raise in anxiety will appear, and consequently there may be no urge to perform compulsions. These processes may occur in acute response to each other, making it impossible to observe a temporal lag between a change in cognitive processes and in OC symptoms [67]. Following this line of reasoning, it may be complicated to get a grip on these fast and dynamic processes.

A third challenge is that mediating processes may differ across individuals. This hypothesis is supported by a study in adult OCD patients on the role of dysfunctional beliefs in CBT [42]. Indeed, there is some evidence that not all OCD patients experience more dysfunctional beliefs than non-clinical individuals [68, 69], and cognitive mediation may not be expected for these patients. In addition, our findings showed a wide range of OBQ-CV scores, indicating that there are substantial individual differences in dysfunctional beliefs, and consequently there may be differences in cognitive change processes during treatment.

Although the present study has several strengths such as a longitudinal design with a mid-treatment assessment, a representative sample of youth with OCD, and the use of a treatment protocol that has already been implemented in clinical practice, the study has some limitations too. First, the present sample size did not allow for adding extra variables to the models, and therefore we could not control for effects of possible moderating variables such as OCD subtype or developmental level. However, a previous study showed no effect of age on OBQ-CV score in a clinical OCD sample (mainly the same sample as the present study) [28]. This finding makes it less likely that results would have been different when age was included. Furthermore, the present treatment protocol allowed for tailoring cognitive interventions to the individual to preclude the risk that these interventions would have been too difficult to understand for younger children, or too childish for older children. This way, we aimed to provide a treatment that suited all participants. Second, due to the time interval between assessment points we may have been unable to detect change processes that have occurred in-between assessment points. Third, reports of dysfunctional beliefs were solely based on a standardized questionnaire. The OBQ-CV was selected because adequate reliability and validity had been demonstrated in pediatric OCD and in a youth community sample [28, 70]. Furthermore, the OBQ-CV includes multiple dysfunctional belief domains assumed to be relevant in OCD, instead of a limited selection of domains [71, 72]. However, despite these strong features of the OBQ-CV, the incorporation of multiple belief domains also entails a disadvantage. Participants who frequently experience beliefs in one domain, may have a relatively low OBQ-CV total score despite the high frequency of particular beliefs. In these cases only small changes can be found, and even if dysfunctional beliefs change during treatment it would be hard to demonstrate mediation effects over time. We have considered to perform analyses using OBQ subscales instead of a general total scale. However, besides that this would have added extra parameters to the model, a study on psychometric properties of the OBQ-CV showed high correlations among subscales, and a single higher-order factor (OBQ total score) that explained the correlations between the subscales quite well [28]. For these reasons, we did not differentiate between subscales. Another complicating factor of using explicit measures is that a certain level of insight in thought processes is required for a valid report of dysfunctional beliefs. This may be demanding for all individuals, and especially for children as meta-cognitive skills are not fully developed at this age. Finally, the main part of the sample was of a Western background. This may limit the generalizability of the results to samples of other cultural backgrounds.

Notwithstanding these limitations, some clinical implications and recommendations for future research can be derived from the present study. Based on the present results, we conclude that restructuring explicit cognitions may not be a necessary component in the treatment of OCD in children and adolescents. At least not for all patients with OCD. For future studies it would be interesting to shed more light on potential moderating variables, for example OCD subtype and children with and without obsessions (tic-related OCD), in combination with mediating variables. Furthermore, idiosyncratic and implicit measures could be combined with standardized, explicit measures of dysfunctional beliefs to examine the role of both explicit and implicit cognitive processes in CBT.

For therapists it would be interesting to know if treatment for pediatric OCD could be confined to solely ERP. The present results do not allow for such a conclusion. First of all, our results need to be replicated. Second, this conclusion would assume a single, one-to-one relationship between CT and a change in dysfunctional beliefs. This seems no realistic representation of matters. We cannot equate CT with cognitive change, and ERP with behavioral change. At present, the actual mechanisms of change of CT and ERP are inadequately understood. Different explicit and implicit processes may be active in CT and ERP, and there may be different ways to achieve treatment effect. Furthermore, in many cases elements of exposure are incorporated in CT in the form of behavioral experiments, and ERP is often complemented by cognitive interventions. The latter is explicitly prescribed in the inhibitory learning model of ERP where expectations about what may happen following exposure are challenged by the therapist [12]. Challenging cognitions is not specifically prescribed in the habituation model of ERP, but this intervention can be used to support ERP [13].

Future studies that aim to evaluate mediating (cognitive) mechanisms in treatment for OCD, should include more in-treatment assessment points. To gain more insight in change processes of dysfunctional beliefs and OC symptoms, these constructs could be measured each session or every day during treatment. Case-based time-series designs can then be used to closely follow changes over time within individuals [42, 45, 46], before resource demanding, large randomized controlled trials are conducted. Although seldom used, case-based time series designs are recognized as fair methodological approaches for treatment studies [73], and can provide information about what interventions work for whom. The latter may be especially important in the light of the large individual differences in treatment effects for pediatric OCD, and because mediating mechanisms may differ across patients.

## Summary

It is generally assumed that explicitly challenging dysfunctional cognitions plays an important role in psychological treatment for OCD. However, to date there hardly is any empirical support for this assumption, especially for pediatric OCD. The aim of the present study was to examine if changing dysfunctional beliefs is a mediator of CBT for pediatric OCD. Fifty-eight children and adolescents (8–18 years; 24 boys) with a primary diagnosis of OCD received sixteen weekly sessions manualized CBT. CBT included exposure and response prevention, and cognitive interventions. Dysfunctional beliefs (OBQ-CV) and OCD severity (CY-BOCS) were measured at different time-points during treatment (pre-treatment, mid-treatment, post-treatment), and at 16-week follow-up. Results showed that both OCD severity and dysfunctional beliefs decreased during CBT. However, contrary to what would be expected from cognitive models, changes in severity statistically predicted changes in dysfunctional beliefs within the same time interval, rather than the reverse. The present results did not support the hypothesis that changing dysfunctional beliefs is a mediating mechanism in CBT for childhood OCD. This finding suggests that restructuring explicit cognitions might not be a necessary component in the treatment of OCD in children and adolescents. Future studies are needed to replicate the present findings and shed more light on the role of explicit and implicit cognitions in CBT for childhood OCD.

## Footnotes

1 Clinical trial registration information: Information processing, neuropsychological, and neurobiological processes in pediatric obsessive–compulsive disorder; http://www.controlled-trials.com/ISRCTN07851536;NTR 717, ISRCTN07851536.

2 Unique variances were calculated by Var(X)*(1- reliability) [57].
